# Multidisciplinary study of a new ClC-1 mutation causing myotonia congenita: a paradigm to understand and treat ion channelopathies

**DOI:** 10.1096/fj.201500079R

**Published:** 2016-06-20

**Authors:** Paola Imbrici, Concetta Altamura, Giulia Maria Camerino, Giuseppe Felice Mangiatordi, Elena Conte, Lorenzo Maggi, Raffaella Brugnoni, Kejla Musaraj, Roberta Caloiero, Domenico Alberga, Renè Massimiliano Marsano, Giulia Ricci, Gabriele Siciliano, Orazio Nicolotti, Marina Mora, Pia Bernasconi, Jean-Francois Desaphy, Renato Mantegazza, Diana Conte Camerino

**Affiliations:** * Department of Pharmacy, Drug Sciences, University of Bari “Aldo Moro,” Bari, Italy;; † Division of Neurology, Neuroimmunology and Neuromuscular Diseases Unit, Istituti di Ricovero e Cura a Carattere Scientifico Fondazione Istituto Neurologico “Carlo Besta,” Milan, Italy;; ‡ Department of Physics “M. Merlin,” Istituto Nazionale di Fisica Nucleare and Centro di Tecnologie Innovative per la Rilevazione e l'Elaborazione del Segnale, University of Bari “Aldo Moro,” Bari, Italy;; § Department of Biology, University of Bari “Aldo Moro,” Bari, Italy;; ¶ Department of Clinical and Experimental Medicine, Section of Neurology, University of Pisa, Pisa, Italy;; ‖ Department of Biomedical Sciences and Human Oncology, University of Bari “Aldo Moro,” Bari, Italy

**Keywords:** chloride channel, gene expression, molecular dynamics, patch-clamp, skeletal muscle

## Abstract

Myotonia congenita is an inherited disease that is characterized by impaired muscle relaxation after contraction caused by loss-of-function mutations in the skeletal muscle ClC-1 channel. We report a novel ClC-1 mutation, T335N, that is associated with a mild phenotype in 1 patient, located in the extracellular I-J loop. The purpose of this study was to provide a solid correlation between T335N dysfunction and clinical symptoms in the affected patient as well as to offer hints for drug development. Our multidisciplinary approach includes patch-clamp electrophysiology on T335N and ClC-1 wild-type channels expressed in tsA201 cells, Western blot and quantitative PCR analyses on muscle biopsies from patient and unaffected individuals, and molecular dynamics simulations using a homology model of the ClC-1 dimer. T335N channels display reduced chloride currents as a result of gating alterations rather than altered surface expression. Molecular dynamics simulations suggest that the I-J loop might be involved in conformational changes that occur at the dimer interface, thus affecting gating. Finally, the gene expression profile of T335N carrier showed a diverse expression of K^+^ channel genes, compared with control individuals, as potentially contributing to the phenotype. This experimental paradigm satisfactorily explained myotonia in the patient. Furthermore, it could be relevant to the study and therapy of any channelopathy.—Imbrici, P., Altamura, C., Camerino, G. M., Mangiatordi, G. F., Conte, E., Maggi, L., Brugnoni, R., Musaraj, K., Caloiero, R., Alberga, D., Marsano, R. M., Ricci, G., Siciliano, G., Nicolotti, O., Mora, M., Bernasconi, P., Desaphy, J.-F., Mantegazza, R., Camerino, D. C. Multidisciplinary study of a new ClC-1 mutation causing myotonia congenita: a paradigm to understand and treat ion channelopathies.

Myotonia congenita (MC), in its dominant and recessive form, is the most common skeletal muscle ion channelopathy and is characterized by muscle stiffness after voluntary effort (1). Usually, recessive MC has an early age of onset and is more severe. Dominant MC encompasses a wider range of symptoms, from subclinical to moderately severe manifestations (1, 2). MC is caused by loss-of-function mutations in the *CLCN1* gene that encodes the voltage-gated chloride channel, ClC-1, which maintains the large shunting chloride conductance of muscle fibers (3).

As for other ion channel diseases, functional studies using heterologous expression systems have been decisive to clarify molecular mechanisms underlying myotonia. Indeed, in a majority of cases, biophysical characterization of expressed ClC-1 mutants satisfactorily demonstrated that specific ClC-1 defects were the obvious culprit for clinical features of patients (4, 5). *In vitro* studies proved that 2 recessive mutations must be present, each affecting the function of 1 allele, to reduce chloride currents sufficiently to produce myotonia (6, 7). In contrast, most ClC-1 dominant mutants exert a dominant-negative effect on the associated wild-type (WT) subunit in the heterodimeric channel (8); however, in some cases, results of functional studies of expressed mutant channels were inadequate to predict a clear correlation between genotype and clinical symptoms, essential to define the most appropriate treatment (9). For instance, myotonic individuals who carry the same ClC-1 mutation can have markedly variable expressivity (10). Some mutations may appear recessive or dominant in different families, whereas others show incomplete penetrance. Several recessive mutations show no evidence of defective function in heterologous systems (5, 11, 12), and some dominant mutations do not induce the expected dominant-negative effect on the WT subunit (4–6). These evidences suggest that, as in the case of other monogenic disorders, environmental factors and genetic background of patients may further contribute to variability of MC phenotype (10, 13). Thus, to fulfill the limits of heterologous expression, we recently studied muscle biopsies from patients with recessive MC to identify possible disease modifiers among genes that are involved in muscle excitability (11), demonstrating that biologic samples from affected individuals can yield valuable information for understanding of the myotonic phenotype. Muscle biopsies can also help achieve a proper diagnosis of myotonia (14).

Another unsolved issue regarding myotonia, which is common to a significant number of ion channelopathies, is lack of a specific therapy. Myotonic patients are usually administered symptomatic treatment, including mexiletine, a sodium channel blocker, or acetazolamide, a carbonic anhydrase inhibitor, with limited side effects and high percentages of nonresponders (15–17). Absence of direct ClC-1 opener prompts identification of novel targets and development of new drugs. In this respect, analysis of patients’ muscle biopsies, together with pharmacologic studies on expressed channels, may support drug discovery. Furthermore, information about the structure-function relationship of mutant channels, gained from the molecular dynamics (MD) simulations of the 3-dimensional (3D) structures of related CLC Cl^−^/H^+^ transporters, may be precious for structure-function studies and for rational design of ClC-1 ligands (5, 18–20).

Here, we report a novel *CLCN1* variant, T335N, that is associated with a mild Thomsen MC phenotype identified in one Italian patient. This mutation resides in the extracellular I-J loop of the ClC-1 protein, whose role in channel function is unknown. We validated a multidisciplinary approach that includes clinical, functional, and molecular analyses by using heterologous expression of WT and mutant ClC-1 channels as well as patient muscle biopsy and MD simulations. Such study allowed us to comprehensively correlate the molecular and functional phenotype with clinical manifestations of the affected patient and to offer a solid starting point to address drug design and therapy. Concerted application of different experimental techniques is thus proposed as a promising paradigm for in-depth understanding of disease mechanisms, which may lead to personalized therapy of this and other ion channelopathies.

## MATERIALS AND METHODS

### Mutagenesis and expression of hClC-1 WT and mutant channels

The c.1004C>A mutation was introduced into the plasmid pRcCMV-hClC-1 that contained the full-length WT hClC-1 cDNA by using the QuikChange site-directed mutagenesis kit (Stratagene, Santa Clara, CA, USA), as previously described (5). The complete coding region of the cDNA was sequenced to exclude polymerase errors. tsA201 cells were transiently transfected with a mixture of hClC-1 WT or T335N (5 μg) and CD8 reporter plasmids (1 μg) by using the calcium phosphate precipitation method. Only cells that were decorated with anti-CD8 antibody–coated microbeads (Dynabeads M450; Thermo Fisher Scientific Life Sciences, Waltham, MA, USA) were used for patch-clamp recordings.

### Electrophysiology and data analysis

Standard whole-cell patch-clamp recordings were performed at room temperature (∼20°C) by using an Axopatch 200B amplifier (Axon Instruments, Sunnyvale, CA, USA) (5). We measured the I-V relationship and overall apparent open probability in high-chloride (134 mM) intracellular solutions to enhance current amplitude and in more physiologic low internal chloride (4 mM) solutions. Composition of the extracellular solution was as follows (in mM): 140 NaCl, 4 KCl, 2 CaCl_2_, 1 MgCl_2_, and 5 HEPES, pH 7.4 with NaOH. High-chloride pipette solution contained (in mM): 130 CsCl, 2 MgCl_2_, 5 EGTA, and 10 HEPES, pH 7.4 with CsOH. In this condition, cells were clamped at the holding potential of 0 mV. In the low-chloride internal solution, cesium chloride was substituted by cesium glutamate and cells were clamped at the holding potential of –95 mV. Pipettes were pulled from borosilicate glass and had ∼2.5 MΩ resistance. Currents were low-pass filtered at 2 kHz and digitized with sampling rates of 50 kHz by using the Digidata 1440A AD/DA converter (Axon Instruments). In high-chloride solution, 400-ms voltage steps were applied from −200 mV to +150 mV in 10-mV intervals, each followed by a 100-ms voltage step at −105 mV, where tail currents were measured. In low-chloride solution, currents were elicited by 400-ms voltage steps between −150 mV and +150 mV in 10-mV intervals, followed by a 100-ms test pulse to −105 mV (5). Instantaneous and steady-state current–voltage relationships were drawn by measuring instantaneous and steady-state current densities (pA/pF) at the beginning (∼1 ms) and at the end (∼390 ms) of each voltage step. Voltage-dependence of channel activation was determined by plotting the apparent open probability (*P*_o_), obtained from normalized peak tail currents at −105 mV, as a function of the voltage of the preceding pulses (5). As saturation of mutant currents at positive potentials was less pronounced than that of WT, maximum current for normalization was arbitrarily taken at +150 mV to calculate overall open probability. Points were fitted with a Boltzmann function:
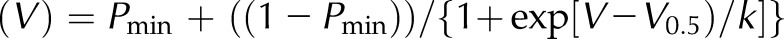
where *P*_min_ is the minimal value of *P*_o_, *V*_0.5_ is the half-maximal activation potential, and k is the slope factor.

Open probability for slow gating (*P*_o_^slow^) was obtained by using a protocol similar to that for the overall *P*_o_ (200 ms conditioning pulse), except that an extra 400-μs activation pulse to +180 mV was added before stepping to −105 mV. This very positive step fully activates the fast gates of the channel and the tail currents at −105 mV, then reflect only slow gating (21). Apparent *P*_o_ and *P*_o_^slow^ were calculated from normalized instantaneous current amplitude that was measured at the beginning of the tail pulse at −105 mV. Because overall apparent open probability equals the product of its fast and slow components, open probability of the fast gates (*P*_o_^fast^) was then calculated for a given test voltage by dividing the relevant *P*_o_ by its corresponding *P*_o_^slow^. Voltage dependence of channel activation was examined by plotting the apparent open probability as a function of membrane potential and fitting data points with a Boltzmann function (5). Data were analyzed by using pClamp 10.3 (Axon Instruments) and Sigma Plot Software (Systat Software, Chicago, IL, USA). Results are reported as means ± sem from *n* cells, and statistical analysis was performed by using Student's *t* test, with *P* < 0.05 considered significant.

### Quantitative real-time PCR analysis on muscle biopsy

All gene expression experiments were performed as previously described in Portaro *et al.* (11). Sections of human muscle biopsies were snap frozen in liquid nitrogen soon after removal and stored at –80°C until use. For each muscle sample, total RNA was isolated by an RNeasy Fibrous Tissue Mini Kit (Qiagen, Valencia, CA, USA) and quantified by using a spectrophotometer (Thermo Fisher Scientific Life Sciences). RT and amplification were performed by Ovation PicoSL WTA system V2 (NuGen, San Carlos, CA, USA). Real-time PCR was performed in triplicate by using the Applied Biosystems real-time PCR 7500 Fast system (Foster City, CA, USA). Each reaction was carried out in triplicate on a single plex reaction. Results were compared with a relative standard curve obtained by 6 points of 1:4 serial dilutions. mRNA expression of different genes was normalized to the best housekeeping gene, β-actin (*ACTB*), selected among β-2 microglobulin (*B2M*), hypoxanthine phosphoribosyltransferase 1 (*HPRT1*), and *ACTB*. For *KCNJ2,* a poorly expressed gene, a preamplification by TaqMan PreAmp Master Mix (Thermo Fisher Scientific Life Sciences) was made before real-time experiments. Real-time PCR experiments were performed in agreement with the MIQE guidelines for quantitative PCR (qPCR), as published previously (22). TaqMan Hydrolysis primer and probe gene expression assays IDs are detailed in the Supplemental Data.

### Western blot analysis of muscle biopsies and transfected cells

hClC-1 was isolated from muscle cells according to Papponen *et al.* (23), with some modifications. In brief, human muscle biopsies were homogenized in ice-cold buffer that contained 20 mM HEPES (pH 7.4), 2 mM EDTA, 0.2 mM EGTA, 0.3 M sucrose, and 0.2 mM PMSF. Homogenates were centrifuged at 7000 *g* for 5 min at 4°C. Supernatant was centrifuged at 50,000 *g* for 1 h at 4°C, and the pellet was solubilized in 20 μl of the same buffer. tsA cells transfected with hClC-1 (10 μg) were harvested in 200 µl of cold RIPA buffer (150 mM NaCl, 0.1% SDS, 1% NP-40, 0.5% Na deoxycholate, 100 mM Na_3_*V*o_3_, 10 mg/ml PMSF, and a protease inhibitor cocktail) and incubated for 10 min in ice. To complete cellular lysis, cell suspensions were passed through a syringe with a needle 10 times. After 15 min of incubation in ice, cell lysates were centrifuged at 14,000 rpm for 30 min at 4°C, and supernatant was collected. Total protein amounts were quantified by using a Bradford protein assay kit (Bio-Rad, Hercules, CA, USA). Proteins (40 μg from muscle biopsies; 1 μg from transfected cells) were separated on a 4–12% SDS-PAGE and transferred onto nitrocellulose membrane for 1 h at 200 mA (SemiDry transferblot; Bio-Rad). Membrane was blocked for 2 h with 0.2 M Tris-HCl, 1.5 M NaCl, pH 7.4 buffer (TBS) that contained 5% nonfat dry milk and 0.5% Tween-20 and incubated overnight at 4°C with rabbit anti–ClC-1 polyclonal antibody (c.n. MBS714620; MyBiosource, San Diego, CA, USA) that was diluted 1:200 with TBS that contained 5% nonfat dry milk. After 3 washes with TBS that contained 0.5% Tween-20, membrane was incubated for 1 h with goat anti-rabbit IgG conjugated to horseradish peroxidase (Sigma-Aldrich, St. Louis, MO, USA). Membrane was then washed with TBS with Tween-20, developed with a chemiluminescent substrate (Clarity Western ECL Substrate; Bio-Rad), and visualized on a Chemidoc imaging system (Bio-Rad).

### Biotinylation of cell-surface proteins

Cell-surface biotinylation was carried out with the Pierce Cell Surface Protein Isolation Kit (Pierce, Rockford, IL, USA). tsA201 cells were transfected with pRcCMV-hClC-1, T335N, or both constructs (10 μg each). One day after transfection, cell-surface proteins were labeled with sulfosuccinimidyl-2-(biotinamido)ethyl-1,3-dithiopropionate (Sulfo-NHS-SS-biotin). In brief, cells were washed with ice-cold PBS twice, and Sulfo-NHS-SS-biotin was added and incubated at 4°C, with constant rotation for 30 min. Excess biotin was quenched with quenching solution. Cells were treated with lysis buffer and centrifuged at 10,000 *g* for 2 min at 4°C. Clear supernatant was reacted with immobilized NeutrAvidin gel slurry in columns to isolate surface proteins. Columns were washed and protein eluted in sample buffer that contained DTT. Surface proteins were separated on a SDS-PAGE gel, and samples (1 μg) were analyzed by Western blotting using a monoclonal anti–ClC-1 antibody (MyBiosource). Filters were also immunoblotted with β-actin monoclonal antibody (Sigma-Aldrich) as control. Total protein density was standardized as the ratio of input signal to β-actin signal, normalized to WT control. Surface protein density was measured as the ratio of surface signal to cognate total β-actin signal, followed by normalization to WT control.

### Molecular dynamics

The homology model of ClC-1, recently published by Bennetts and Parker (24), was used as initial structure. It consists of the ClC-1 dimer that is based on the crystallographic coordinates of CmClC [Protein Data Bank (PDB) ID 3ORG; *http://www.rcsb.org/pdb/*] (19). Following the protocol of a recent work (5), we excluded from the model the C-terminal (from residue 588). This enabled us to perform 2 independent long MD simulations (2 × 50 ns) for each investigated system with a reasonable computational time. As a first step, we pretreated the resulting structure by using the protein preparation wizard (version 9.5; Shrödinger, New York, NY, USA) (25). Simulation system was built as follows: A 110 × 140 Å^2^ POPC (1-palmitoyl,2-oleoyl-*sn*-glycero-3phosphocholine) bilayer patch was first built by using the membrane plugin of visual molecular dynamics (VMD) (26), with the membrane normal along the *z* axis. ClC-1 dimer was embedded in this bilayer, and lipid molecules within 0.6 Å of heavy atoms of the protein were removed. To neutralize the system, 47 Na^+^ and 65 Cl^−^ ions were added using the VMD autoionize plugin, generating a 150-mM ionic concentration and a final system of 136,784 atoms (number computed for WT). Two different protein structures were built, the WT form and the mutated T335N form. The latter was obtained by using the mutator plugin available in VMD. Both T335N and WT protein structures were incorporated into a periodic box of TIP3P water molecules (27) that was extended by 18 Å in each direction from all protein atoms by using the Add Solvation Box plugin of VMD. All MD simulations were performed using NAMD 2.9 (28) and the CHARMM27 force field (29). Note that the useConstantArea option was set to on to keep realistic lipid structures. The full system was minimized to remove steric clashes in the initial geometry and was gradually heated up to 310 K within 500 ps of MD. SHAKE algorithm was employed to constrain all R–H bonds (30). Rigid water molecules were implemented by using the SETTLE algorithm (31). Periodic boundary conditions were applied in all directions. A nonbonded cutoff of 12 Å was used, whereas the Particle-Mesh-Ewald (32) was employed to include contributions of long-range interactions. All simulations were performed in an isothermal-isobaric ensemble (1 atm, 310 K) with a modified Nosé–Hoover method in which Langevin dynamics was used to control fluctuations in the barostat, as implemented in NAMD 2.9 (33) (oscillation period, 200 fs; decay coefficient, 100 fs), and a Langevin thermostat (34) (damping coefficient, 1 ps^−1^). Time step was set to 2 fs, and coordinates were saved every 5000 steps (10 ps). Two MD trajectories of 50 ns were obtained for both the WT and the mutated T335N form. For each investigated system, equilibration of the structure required <5 ns and, thus, we removed the early 5 ns from analysis. To visualize the ClC-1 3D structure and the position of the mutated residue, the homology model was rendered by using PyMOL (Shrödinger).

## RESULTS

### Clinical phenotype

A single c.1004C>A mutation in exon 9 of the *CLCN1* gene, corresponding to p.T335N amino acid substitution in ClC-1, was detected in one Italian patient. The proband is a 43-yr-old male who presented at age 15 yr with lower-limb muscle stiffness, sometimes painful and mainly occurring at the beginning of movement after prolonged rest and improving with exercise. The patient has never complained of cold-aggravated myotonia or transient weakness. Neurologic examination revealed myotonia with warm-up, mainly in quadriceps without muscle weakness or hypertrophy. Electromyography showed myotonic discharges in all investigated muscles. Symptoms remained stable over the years without any relevant limitation in daily activities. The patient has never taken any treatment for myotonia. Except for absence of muscle hypertrophy, clinical phenotype was compatible with Thomsen disease. Family history was negative.

### Functional analysis of T335N channels expressed in tsA201 cells

**Figure 1.**
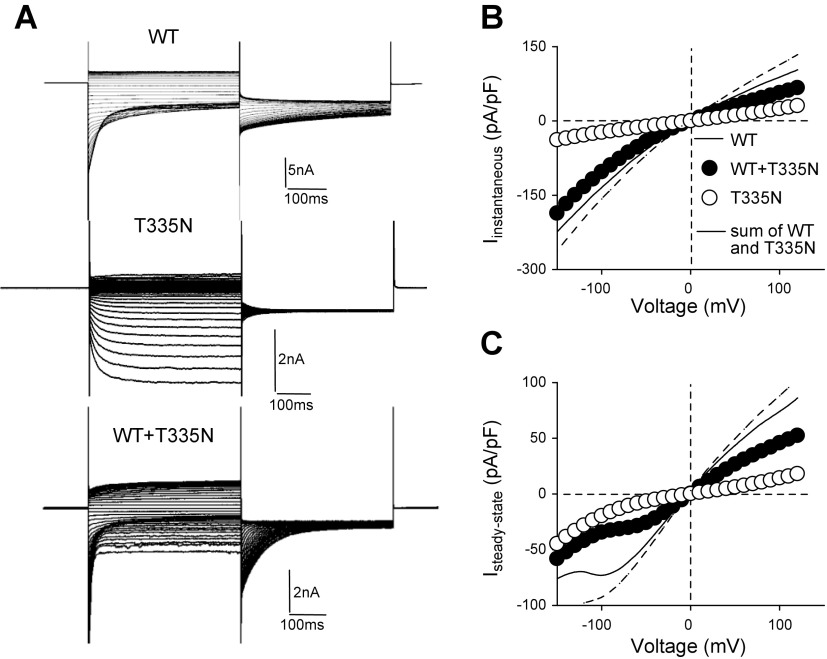
Current amplitude for hClC-1 WT, T335N, and WT + T335N channels in high intracellular chloride. *A*) Representative chloride currents recorded from tsA cells transfected with hClC-1 WT (5 μg), T335N (5 μg), or with equal amount of WT and T335N (5 μg + 5 μg) cDNAs in high-chloride intracellular solution. *B*) Instantaneous currents were normalized with respect to cell capacitance (pA/pF) and reported as a function of voltage. *C*) Steady-state currents were reported as mean current density as a function of voltage. Solid lines represent WT currents; dashed line represents the algebraic sum of current densities calculated for WT and T335N expressed alone. Each point is mean ± sem from 13–18 cells.

**Figure 2.**
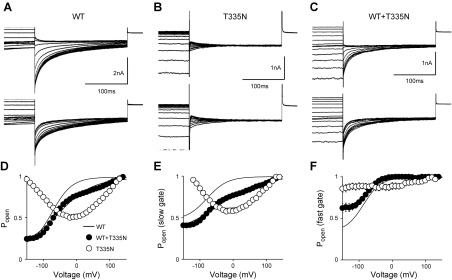
Open probability for hClC-1 WT, T335N, and WT + T335N hClC-1 channels in high intracellular chloride. *A*–*C*) Representative tail current traces elicited from tsA cells transfected with WT (*A*), T335N (*B*), and WT+T335N (*C*) from experiments to determine overall gating (top) and slow gating (bottom) in high intracellular chloride solution. *D*–*F*) Apparent *P*_o_ for overall (*D*), slow (*E*), and fast gating (*F*) plotted as a function of test voltage, for WT, T335N, and WT + T335N channels. Relationships obtained from averaged data were fit with a Boltzmann equation. Solid lines represent the WT currents. Open probability for slow gating was obtained as described in Supplemental Data. Fit parameters are reported in Tables 1 and 2. Each point is mean ± sem from 7–11 cells.

Mutation T335N is associated with a mild Thomsen phenotype and resides in the extracellular loop that connects helices I and J (Supplemental Fig. 1). To test whether this conserved amino-acidic variant altered ClC-1 permeation and gating to cause myotonia in the patient, we transfected tsA201 cells with equal amounts of WT or T335N cDNA and analyzed chloride currents by whole-cell patch-clamp. As shown in **Fig. 1*A***, in high intracellular chloride solution, T335N homodimeric channels showed a gating behavior very different from that of WT. Indeed, T335N currents activated slowly at hyperpolarized potentials and lacked fast deactivation typical of WT ClC-1 currents. Instantaneous and steady-state current densities for T335N were reduced at each voltage compared with WT (Fig. 1*B*, *C* and **Table 1**). The attempt to fit normalized tail currents as a function of voltage with a Boltzmann function revealed an ∼160 mV rightward shift of voltage-dependent activation for T335N with respect to WT (**Fig. 2*A*, *B*, *D*** and Table 1). As hClC-1 is a dimer formed by 2 identical subunits that open and close through a fast or slow gating pattern (21), we also analyzed the effect of the T335N mutation on the open probability for fast and slow gating. Of interest, voltage dependence for slow gating was shifted by about ∼160 mV in the positive direction, whereas fast gating was abrogated (Fig. 2*B*, *E*, *F* and **Table 2**).

**TABLE 1.** Biophysical parameters for hClC-1 WT, T335N, and WT + T335N channels

ClC-1	[Cl−] (mM)	*V*_0.5_ *P*_o_^total^[mV (*k*)]	*P*_min_^total^	IC (pA/pF)	SSC (pA/pF)	Cells (*n*)
WT	134	−74 ± 1 (30 ± 1)	0.45 ± 0.04	−72 ± 6.7	50.5 ± 6	15
T335N	134	94 ± 5* (25 ± 2)	0.68 ± 0.06	−13 ± 2.9*	7.6 ± 2.2*	13
WT + T335N	134	−50 ± 3 (51 ± 2)	0.39 ± 0.03	−59 ± 6	31 ± 3.8	18
WT	4	−22.9 ± 1.3 (37 ± 1)	0.23 ± 0.03	—	35 ± 8.9	8
T335N	4	39.1 ± 4.4 (72 ± 5)*	0.45 ± 0.03*	—	5.8 ± 1*	10
WT + T335N	4	30.1 ± 6.7 (74 ± 8)*	0.17 ± 0.03	—	17.9 ± 6.3*	7

Data are means ± sem of the indicated number of cells. *V*_0.5_, half maximal activation potential; *k*, slope factor; *P*_min_, minimal value of *P*_o_ measured at −90 mV; IC, instantaneous current measured at −60 mV; SSC, steady state current measured at +60 mV. *P < 0.05 compared with WT.

**TABLE 2.** Open probability for slow and fast gating for hClC-1 WT, T335N, and WT + T335N channels in high-chloride solution

ClC-1	*V*_0.5_ *P*_o_^slow^[mV (*k*)]	*P*_min_^slow^	*V*_0.5_ *P*_o_^fast^[mV (*k*)]	*P*_min_^fast^	Cells (*n*)
WT	**−**79 ± 2 (31 ± 1)	0.69 ± 0.03	**−**87 ± 1 (27 ± 1)	0.64 ± 0.04	10
T335N	90 ± 7 (31 ± 3)*	0.78 ± 0.05	—	0.89 ± 0.07	7
WT + T335N	**−**40 ± 4 (58 ± 3)*	0.53 ± 0.03	**−**76 ± 1 (18 ± 1)	0.72 ± 0.02	11

Data are means ± sem of the indicated number of cells. *V*_0.5_, half maximal activation potential; *k*, slope factor; *P*_min_, minimal value of *P*_o_ measured at −90 mV; *P*_o_^fast^, open probability of the fast gate; *P*_o_^slow^, open probability of the slow gate. *P < 0.05 compared with WT.

To have a clearer picture of the functional behavior of homomeric T335N channels, the same biophysical characterization was performed under more physiologic recording conditions. Of note, in low intracellular chloride solution, hyperpolarization-induced activation was abolished, current was significantly reduced, and *P*_o_ was right shifted for T335N compared with WT channels (Supplemental Fig. 2*A*–*C* and Table 1), corroborating previous results.

As the affected patient carries 1 normal and 1 mutated *CLCN1* allele, the major contribution to total chloride current is plausibly represented by heterodimeric WT + T335N channels, assuming that WT and T335N subunits are equally expressed. Thus, to better reproduce *in vitro* this heterozygous condition, we coexpressed T335N with equal amounts of WT cDNA in tsA cells. Of interest, in high-chloride solution, WT + T335N channels showed gating properties similar to WT, with both inwardly and outwardly rectifying currents but without the hyperpolarization-activated component (Fig. 1*A*). The steady-state current density of heteromeric channels was, at each voltage, less than half of that of homomeric WT channels (Fig. 1*C*). In addition, total *P*_o_ was shifted by ∼30 mV toward more positive potentials compared with respective WT homomeric channels (Fig. 2*C*, *D* and Table 1), so that WT + T335N channels were less likely to be open in the physiologic voltage range. Separation of common and fast gating revealed that voltage dependence for slow and fast gating was significantly shifted by approximately +40 and +10 mV, respectively (Fig. 2*E*, *F* and Table 2). Of interest, patch-clamp recordings of WT + T335N channels carried out in low-chloride intracellular solution confirmed biophysical alterations observed in high intracellular chloride solution. Indeed, we detected a positive shift of WT + T335N total *P*_o_ and a dramatic reduction of steady-state current compared with homomeric WT channels (Supplemental Fig. 2*A*–*C* and Table 1). This dominant effect of the mutant on the WT subunit reasonably contributed to the decline in sarcolemmal gCl in the affected carrier.

**Figure 3.**
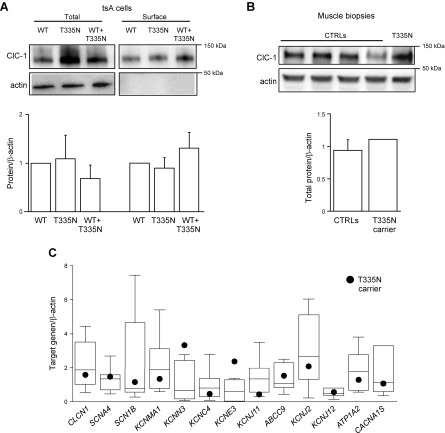
Quantification of protein amounts and analysis of gene expression. *A*) Western blot of total and surface ClC-1 and β-actin proteins from tsA cells transfected with equal amounts of WT (10 μg), T335N (10 μg), and WT + T335N (5 μg + 5 μg; top) and quantification of total and surface proteins normalized to WT (bottom). Each bar is mean ± sem from 3 experiments. *B*) Western blot (top) and quantification (bottom) of total ClC-1 and β-actin proteins in muscle tissue from 4 control individuals (CTRLs) and T335N patient. *C*) Quantitative gene expression of selected ion channels and transporters from vastus lateralis muscle biopsies of control individuals and T335N patient. For each gene, box plot encloses 50% of data from 6 controls, with the median value displayed as a line. Closed circles represent T335N; top and bottom of the box mark the limits of ±25% of the range of data; lines extending from the top and bottom of each box mark the minimum and maximum values within the data set that fall within an acceptable range. Data are means of triplicate measures.

Reduction of chloride current observed when mutant channels were expressed in tsA cells could be accounted for by an alteration of gating and/or by a reduction in the number of functional channels in the plasma membrane. Thus, to investigate the membrane trafficking property of T335N channels, we performed protein biotinylation assay on cells that expressed WT, T335N, and WT + T335N channels. **Figure 3*A*** shows that T335N channels displayed cell-surface expression similar to that of WT. In addition, coexpression of T335N with WT did not affect membrane trafficking of ClC-1 channels.

### Western blot and quantitative analysis of gene expression in muscle biopsy of patient with T335N

To study T335N channel properties in a more physiologic setting, we next evaluated the amount of total ClC-1 proteins extracted from vastus lateralis muscle biopsies of the T335N carrier and of 4 control individuals. As shown in Fig. 3B, Western blot analysis revealed that total ClC-1 protein amount was similar in controls as well as in the affected patient, which reasonably excluded that alterations in ClC-1 protein synthesis as a result of T335N mutation may contribute to myotonia and supported results obtained from transfected cells.

To assess whether a variation in expression of other genes could further concur to clinical phenotype of the patient and to identify potential drug targets, we next performed a quantitative real-time PCR analysis of a number of gene transcripts from the same muscle biopsy of the proband and compared results with 6 controls of the same gender and age. Genes under investigation encoded for sarcolemmal ion channels and transporters that are involved in action potential generation and propagation or in calcium homeostasis, and/or have been linked to skeletal muscle excitability disorders (Supplemental Table 1). In agreement with Western blot analysis, quantitative PCR analysis showed that the amount of ClC-1 T335N mRNA was within range of control individuals (Fig. 3*C*). Furthermore, we observed no substantial differences between the myotonic patient and controls in the amount of ion channel transcripts, such as voltage-dependent Na^+^ channel hNav1.4 (*SCN4A*), Na^+^ channel β1 subunit (*SCN1B*), large-conductance Ca^2+^-activated K^+^ channel (*KCNMA1*, Slo1), voltage-dependent K^+^ channel Kv3.4 (*KCNC4*), ATP-sensitive, inward rectifier K^+^ (KATP) channel Kir6.2 (*KCNJ11*), sulfonylurea receptor type 2A (SUR2A protein, *ABCC9* gene), 2 inward rectifier K^+^ channels, Kir2.1 (*KCNJ2*) and Kir2.6 (*KCNJ18*), Na^+^/K^+^- ATPase (*ATP1A2*), and Ca^2+^ channel Cav1.1 (*CACNA1S*). The most interesting observation, to be confirmed in other myotonic cases, was the presence of the transcript of MinK-related peptide 2 (*KCNE3*) in this dominant MC biopsy (Fig. 3*C*) that was reported instead to be absent in 2 biopsies of patients with recessive MC (11). In addition, we observed a greater amount of mRNA that encoded the small-conductance Ca^2+^-activated K^+^ channel (*KCNN3*) in the affected patient with respect to controls.

### MD simulations of WT and T335N channels

**Figure 4.**
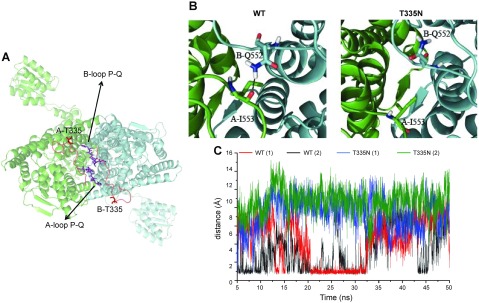
Visual inspection of the ClC-1 homology model and analysis of H-bond interactions. *A*) Top view of the homology model. Atoms belonging to residue T335 and to loop P-Q are rendered as sticks, whereas the ClC-1 dimer is shown in cartoon representation. *B*) Enlarged monomers are depicted in cartoon representation, whereas important residues are shown as sticks. H-bond interaction is depicted by a dotted line. *C*) Time-dependent evolutions of the distance between the nitrogen donor of the side chain of B-Q552 and the oxygen atom of the A-I553 backbone resulting from 2 independent MD simulations of WT (red and black lines) and T335N (blue and green lines).

T335N mutation is located in an extracellular loop that connects helix I and helix J, close to the interface between the 2 monomers (**Fig. 4*A***). To provide a putative molecular explanation for the gating alteration induced by T335N, we carried out an in-depth analysis of obtained MD trajectories for WT and T335N channels. As a first step, we analyzed hydrogen-bond (H-bond) interactions that occurred in the dimer within simulated trajectories, an approach already proven to give valuable insight into the gating mechanisms of ClC-1 and other proteins (5, 35, 36). Of interest, the sole appreciable difference was observed for the extracellular loop that connected helices P and Q, namely, the protein segment from residue in position 551 to that in position 555 (Fig. 4*A*). Such protein portion, which is located close to the loop where mutation takes place, is responsible for intermonomeric interactions at the extracellular side of the dimer. Rate of occurrence (percent) of intermonomeric H-bonds that involve such loop during the entire analyzed trajectories is reported in **Table 3** and shows the percentage of frames in which a given H-bond is formed, using as thresholds a distance between atom acceptor (AA) and atom donor (AD) equal to 3 Å and an angle AD-H–AA equal to 150°. All possible interactions between monomer A and B involved residues Q552 and I553. We observed that the occurrence of such H-bond interactions was far less frequent after mutating the threonine residue in position 335. Of importance, such a trend is observed to account for both analyzed independent trajectories. A meaningful example is given by H-bond interaction that occurrs between Q552 and I553, whose percentage of formation drops down from 16.24% (WT 1, first simulation of WT form) and 16.16% (WT 2, second simulation of WT form) to 0.02% (T335N 1) and 0.00% (T335N 2). Undoubtedly, such H-bond was forthwith lost during MD simulations of T335N. For clarity, Fig. 4*B* shows representative MD snapshots that indicate the residues engaged in this H-bond interaction. As shown in Fig. 4*B*, H-bond interactions could facilitate molecular interplay between the 2 monomers by increasing their proximity. Such a different behavior between WT and T335N was also clearly confirmed by the time dependence of the distance between the H-bond donor and H-bond acceptor side chain atoms of B-Q552 and A-I553 interaction (Fig. 4*C*). Of importance, distance values, which are compatible with H-bond interaction, were never experienced during the 2 simulations of T335N. On the contrary, this interaction occurred frequently during both simulations of WT and, whenever lost, it was re-established after a few nanoseconds. This strongly supported the robustness of the molecular hypothesis that resulted from analysis of the occupancies (Table 3): the replacement of a threonine with a asparagine in position 335 in the I-J loop could alter the intermonomeric interactions that involved the extracellular loop that connects helices P and Q.

**TABLE 3.** Analysis of H-bond interactions

H-bond		Occupancy (%)			
Donor	Acceptor	WT 1	WT 2	T335N 1	T335N 2
B-Q552 side	A-I553 backbone	16.24	16.16	0.02	0.00
A-Q552 side	B-Q552 side	4.64	5.49	2.20	0.64
B-Q552 side	A-Q552 side	3.80	9.24	0.36	0.02
A-I553 backbone	B-Q552 side	5.89	0.00	0.02	0.00

Computed occupancies (%) of the H-bonds occurring between the 2 monomers (A and B) and involving the P-Q loop. An AD–AA distance equal to 3 Å and an angle AD-H–AA equal to 150° were used as thresholds to define the presence of the H-bond.

## DISCUSSION

Here, we performed an in-depth genotype-phenotype correlation of a novel case of MC in which the patient carried the T335N mutation, and we have attempted to provide suggestions for drug development by using a coherent and multidisciplinary experimental plan.

### T335N ClC-1 mutation promotes effects on channel gating that explain myotonia

Patch-clamp recordings of homomeric and heteromeric T335N channels expressed in tsA cells revealed pronounced changes in voltage-dependent gating compared with WT. Indeed, both in high- and low-chloride intracellular solutions, homomeric T335N channels showed dramatically reduced current density, together with a positive shift of voltage dependence of activation. Of importance, chloride currents produced by spontaneous assembly of T335N and WT subunits were smaller than those of WT alone, which indicated a dominant effect of T335N on the WT subunit—an effect typical of most dominant mutations (9). Actually, surface expression of T335N proteins was comparable to that of WT channels alone and T335N subunit did not affect membrane transport of coexpressed WT subunits. To substantiate these biophysical and biochemical data from transfected cells and better explain the clinical phenotype in a more physiologic and reliable setting, we used the available biopsy from the vastus lateralis muscle of the patient to perform gene and protein expression analyses. Of interest, the amount of ClC-1 mRNA and protein detected in the bioptic sample of the T335N carrier was comparable to that of controls. Thus, combination of the biophysical characterization of mutant channels with molecular data obtained from transfected cells and patient biopsy allowed us to reasonably conclude that gating dysfunction of mutant ClC-1 channels might be the principal cause of reduced chloride current and consequent myotonia in this patient.

### Variable expression of ion channel genes in MC

As with many genetic disorders, range of symptoms associated with myotonia might result from interplay of different genes and life conditions that act as disease modifiers (11, 37–41). Muscular dystrophy, sodium channel myotonia, and several forms of idiopathic epilepsies, for example, are associated with variable outcomes that arise from both primary genetic mutation and contribution of environmental and genetic modifiers (37–39) that can influence penetrance, age of onset, progression, or severity of disease (41). In the case of MC, gene expression quantification on muscle biopsies of affected and control individuals could be a valuable and innovative approach for identification of such modifiers. Indeed, in a recent study, quantitative PCR analysis of muscle biopsies from 2 patients who suffered a severe Becker phenotype showed a greater expression of the sodium channel β1 accessory subunit (*SCN1B*) and total lack of the Kv3.4 potassium channel accessory subunit MinK-related peptide 2 (*KCNE3*) (11). Surprisingly, the amount of *KCNE3* and *KCNN3* transcripts in the available Thomsen T335N myotonic muscle fell outside the ranges quantified for control individuals. Although quantification of ion channel transcripts was performed in only 1 patient with MC, these results led us to speculate that increased expression of *KCNE3* and *KCNN3* might modulate the myotonic phenotype in this patient. Neurophysiologic examination of the T335N patient, indeed, reported only mild clinical myotonic signs that do not interfere with everyday life activities. It is worth noting that the poor number of human samples analyzed to date does not allow us to draw a definitive conclusion, and quantitative PCR screening on a larger number of cases is required to validate these results. If confirmed in other patients, these data from muscle biopsies might also suggest alternative pharmacologic targets in myotonia (11). Indeed, proper stimulation of K^+^ currents to compensate for reduced chloride conductance may be explored for treatment of MC. Accordingly, activation of K^+^ currents after carbonic anhydrase inhibition provided a sound explanation for the beneficial action of acetazolamide and its derivatives, which, until now, have been usedonly empirically as antimyotonic drugs (42).

### Structural insight into I-J loop involvement in ClC-1 channel function

Biophysical characterization of naturally occurring mutations in ion channels, together with availability of high-resolution 3D structures of these proteins, can provide relevant information about their structure-function relationship. This, in turn, is pivotal for addressing selective drug design. In the case of T335N mutation, residue T335 is located in the extracellular I-J loop of ClC-1, whose contribution to ClC-1 activity has never been investigated before. From visual inspection of the 3D structure of ClC-1dimers, this loop connects the 2 antiparallel halves (B–I and J–Q) of a ClC-1 monomer (18) and makes contact with the extracellular P-Q loop, which is responsible for interactions between the 2 monomers at the extracellular side. Consistently, the dominant-negative effect of the T335N subunit on the WT observed in patch-clamp experiments also suggests that the I-J loop may affect intermonomeric interactions at the dimer interface (8, 43). Moreover, one peculiar characteristic of T335N homomeric channels in high-chloride solution was the unusual current activation at negative potentials, together with a disruption of fast and slow gating, whose physiologic significance and molecular mechanism are as yet unclear. In this context, combination of MD simulations and electrophysiology elucidated at molecular levels both the functional defect of T335N channels and the role of the I-J loop in ClC-1 channel function. Of note, analysis of MD trajectories of homomeric T335N and WT channels actually demonstrated that the I-J loop might remotely control conformational changes that occur at the boundary region between 2 monomers. In particular, occurrence of the T335N mutation in the I-J loop causes a weakening of the H-bond interaction between residues Q552 and I553 of the P-Q loop of 2 distinct monomers. One possible interpretative hypothesis could be that, as a consequence of this impaired interaction between the 2 T335N subunits, chloride currents lose fast deactivation at negative potentials that is typical of ClC-1 WT channels observed in patch-clamp recordings of T335N channels. Of interest, a hyperpolarization-activated gating, which occurs *via* a cooperative conformational change of 2 mutant subunits, has been previously shown for other heterozygous MC mutations, including Q552R of loop P-Q (44–48). Thus, similar to T335N, activation at negative voltages of Q552R homomers could likely be ascribed to disruption of the intermonomeric H-bond between Q552 and I553.

As T335N channels impaired fast and slow gates, we may also hypothesize that the I-J loop might take part in complex conformational rearrangements that contribute to these gating processes (5, 24, 49, 50). In agreement with the relevance of the I-J loop in ClC-1 activity, it is worth reporting that this loop is involved in the mechanism that underlies niflumic acid and Ca^2+^ potentiation of kidney ClC-K currents (51). Thus, at least in ClC-K channels, this loop might also represent an ideal target for drugs that are aimed at increasing chloride currents. Given the structural similarity within the CLC family (18), it would be appealing to employ such results for addressing the rational design of ClC-1 opener.

In summary, patch-clamp studies of ClC-1 mutants demonstrated that T335N exerts a dominant effect on the WT subunit that likely accounts for myotonia. MD simulations support the electrophysiologic data and envisage the role of the I-J loop in channel gating and modulation after intermonomeric interactions. Quantitative analyses of gene expression in this and previous studies (11) suggest that variations in expression of K^+^ channel genes might contribute to the myotonic phenotype and offer novel druggable targets. This multidisciplinary approach, here validated for MC, could be extended to other channelopathies to allow a better understanding of these disorders and to build the basis for personalized therapy.

## Abbreviations

3D: 3-dimensional
AA: atom acceptor
AD: atom donor
H-bond: hydrogen bond
MC: myotonia congenita
MD: molecular dynamics
*P*_o_: open probability
qPCR: quantitative PCR
TBS: Tris-buffered saline
VMD: visual molecular dynamics
WT: wild-type
